# The Role of Molecular Tests in the Diagnosis of Disseminated Histoplasmosis

**DOI:** 10.3390/jof6010001

**Published:** 2019-12-18

**Authors:** Izadora Clezar da Silva Vasconcellos, Daiane Flores Dalla Lana, Alessandro C. Pasqualotto

**Affiliations:** 1 Post-graduation Program in Pathology, Federal University of Health Sciences of Porto Alegre (UFCSPA), Porto Alegre 90050-170, Brazil; izavasconcellos@hotmail.com (I.C.d.S.V.); dayalana@hotmail.com (D.F.D.L.); 2 Molecular Biology Laboratory, Santa Casa de Misericórdia de Porto Alegre. Av Independência 155, Hospital Dom Vicente Scherer, heliponto, Porto Alegre 90020-090, Brazil

**Keywords:** AIDS, histoplasmosis, immunological tests, molecular diagnosis, PCR

## Abstract

Histoplasmosis is an emerging fungal disease, with global distribution. The disseminated form of the disease is a more severe infection, generally associated with AIDS. Classic diagnostic methods for histoplasmosis consist of microscopy, culture, and histopathology. More recently, the importance of *Histoplasma* antigen detection has dominated the literature on histoplasmosis diagnosis, but the relevance of molecular assays has not been as much studied. Here we describe the results of a systematic literature review focusing on studies that mainly compared immunological techniques (*Histoplasma* urine antigen detection) with molecular tests for the diagnosis of histoplasmosis. In addition to the review of comparative studies using such diagnostic techniques, the literature on polymerase chain reaction (PCR) tests in patients with disseminated histoplasmosis is also summarized. Two studies reported the comparison between immunological and molecular methods applied simultaneously for the diagnosis of disseminated histoplasmosis. PCR demonstrates a satisfactory performance assisting in the detection of *Histoplasma* spp. DNA in clinical samples.

## 1. Introduction

Histoplasmosis is a global emerging fungal disease, with most cases reported in the American continent. The disease is the most common cause for hospitalization and death among the endemic mycoses in the U.S. [1,2]. In Brazil, 3530 patients were diagnosed with histoplasmosis between the years 1939 and 2018, which is likely to be underestimated. Histoplasmosis is endemic in all Brazilian regions, particularly in the Northeast, Central-West, Southeast, and South [3].

Histoplasmosis mainly affects the lungs from which it can disseminate to other body sites such as the adrenal glands, bone marrow, gastrointestinal tract, joints and the brain [2]. Disseminated histoplasmosis (DH)–the more severe and common form of the disease–generally occurs in AIDS patients, in addition to immunocompromised person on treatment with steroids, presenting with severe neutropenia as a result of cancer chemotherapy, as well as recipients of hematopoietic stem cell and solid organ transplantation [2,3].

Classical diagnosis of DH relies on histopathology, microscopy and fungal culture. However, these methods are known for their limited sensitivity, in addition to the prolonged time for culture to reveal results [4,5]. Over the last decade, several studies have shown *Histoplasma* antigen detection to be the main non-invasive test to diagnose such conditions [6,7]. Detection of circulating *Histoplasma* antigens in urine has proven to be highly sensitive (95%), but this methodology still has some limitations, such as cross-reactivity with other dimorphic fungi [8,9]. Conversely, in many countries where DH is endemic, *Histoplasma* antigen test is commercially not available, which opens a field of opportunities for other diagnostic tests to be used in association or in substitution for antigen detection [8,10].

Molecular tests are routinely used in many clinical centers to detect a variety of pathogens, including fungi. In this context, several articles have documented the performance of molecular tests (particularly PCR [polymerase chain reaction]) in DH patients. However, assays vary largely in terms of sequence of probes and primers, in addition to DNA extraction methods and tests platforms [11,12,13]. The diagnostic performance of molecular tests in patients for whom *Histoplasma* antigen detection is also available has not been properly explored. In view of this, the aim of this study was to compare the performance of molecular tests in the diagnosis of DH considering two main scenarios, related to the presence or the absence of *Histoplasma* antigen detection.

## 2. Methods

A PubMed systematic literature review was conducted using the following search strategy: “(histoplasmosis or histoplasma) and (diagnosis or sensitivity or specificity or performance or accuracy) and ((molecular or PCR or sequencing) and (antigen or antigenuria or ELISA))”. The literature review was built using the classical PICO structure, in which “P” stands for “population/patients”; “I” for “intervention”; “C” for “comparison/control”; and “O” for “outcome”. No language or date restriction was applied. References from selected articles were also reviewed. Publications describing the diagnosis of the disease by other methods (i.e., non-molecular) were excluded from the review, as well as studies involving histoplasmosis in non-humans.

## 3. Results

**Figure 1:**
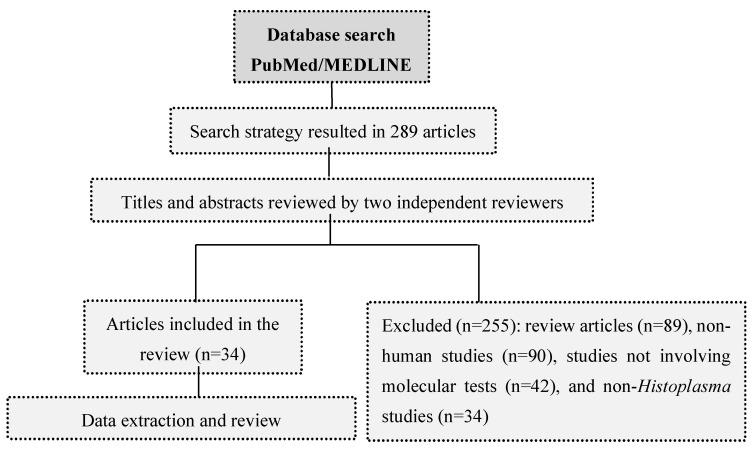
Flowchart with data selection and analysis methods used in the systematic review.

Figure 1 shows the flowchart with data selection and analysis methods used in the study. A total of 289 articles were found in the search. After exclusion of non-appropriate manuscripts, 34 publications were selected to review. From these publications, only two studies directly compared the use of molecular tests with *Histoplasma* antigen detection, in patients with DH. We summarize below the evidence regarding the use of molecular tests for the diagnosis of histoplasmosis.

### 3.1. Comparison of Molecular and Immunological Diagnosis of DH

One of the studies comparing molecular tests with *Histoplasma* antigen assays was performed using urine samples [14]. The study evaluated the performance of a PCR-enzyme immunoassay (PCR-EIA) test against three comparators: two groups with variable concentrations of *Histoplasma* antigenuria and a negative control group (healthy volunteers) [14]. Twelve *H. capsulatum* and *Blastomyces dermatitidis* isolates were chosen to assess the pattern of recognition of the PCR primers. The MiraVista^®^ test was used to detect *Histoplasma* antigen, fungal cultures were incubated for 8 weeks, and the molecular test targeted a 99-bp portion of the *H. capsulatum* 100-kDa protein gene. From 51 samples known to be positive by Miravista^®^ test, only 5 (9.8%) were positive by culture and 4 (7.8%) were detected in the PCR-EIA test. Positive PCR results in urine specimens correlated well with positive culture results, but not with antigenuria.

In another publication, an HIV-infected patient with pulmonary histoplasmosis was reported for presenting a false-positive result with serum *Aspergillus* galactomannan testing [15]. Amplification and sequencing of the ITS1, 5,8S and ITS4 regions of ribosomal DNA, made directly from lung biopsy using panfungal primers, allowed for *H. capsulatum* identification. Mycological cultures turned positive after three weeks, with the presence of numerous thick-walled, echinulate macroconidia, typical of the *Histoplasma* genus, confirming the diagnosis of pulmonary histoplasmosis, primarily detected by the molecular (sequencing) method. The study addressed the potential interest of the *Histoplasma* DNA for the diagnosis of histoplasmosis, especially in endemic countries where *H. capsulatum* antigen testing may not be available.

### 3.2. Overview of the Performance of Molecular Methods in the Diagnosis of DH

There are several factors that influence the reliability and reproducibility of molecular tests, including the number and the type of samples tested. A summary of all studies in which molecular methods were used to diagnose histoplasmosis is presented in Table 1. Fungal culture is one of the best-performing specimens but this has little applicability in the routine diagnostic laboratory, since 4 to 6 weeks are required for fungal growth before PCR application [13]. Most studies in the literature evaluated the performance of nested PCR in the diagnosis of DH, a method that is associated with the increased risk of amplicons contamination, since it involves the manipulation of amplified products. Many of the PCR tests for histoplasmosis were developed in-house [11,12,13,16,17,18,19,20,21,22,23,24,25,26,27]. The gene encoding a 100 kDa molecular protein, which is described as necessary for the survival of *H. capsulatum* in human cells, has been the most common target for the diagnosis of histoplasmosis [11,12,16,19,23,24,27]. Hcp100 is part of the p100 gene family, and it is expressed in large quantities during fungal macrophage invasion [12]. This marker was first tested and standardized in a nested PCR test, with a reported sensitivity of 100% and a specificity of 95% [16,19]. However, as already stated nested PCR is a method that involves great care because of increased risk for contamination, since it involves the manipulation of amplified products.

## 4. Discussion

In this literature review, we were able to find only two studies in which molecular tests were used in parallel to *Histoplasma* immunological antigen detection for DH diagnosis [14,15]. One of the studies [14] reported a very low sensitivity for PCR using urine samples. Urine was tested in the study because this is considered the optimal sample for *Histoplasma* antigen detection using EIA. However, with regard to PCR, urine is usually seen as a clinical material that contains many potential interferents with the PCR reaction. Additional points must also be considered: (i) some false-positive may have occurred in the *Histoplasma* antigen test, since this is known to cross-reacts with *Blastomyces dermatitidis*, also endemic in the U.S.; also, (ii) primers may not have allowed for the detection of all variants of *H. capsulatum* with satisfactory sensitivity. In any case, the study does not tell us about the performance of *Histoplasma* PCR in additional clinical samples, such as whole blood, bone marrow aspirate, bronchoalveolar lavage (BAL) fluid, and tissues. Besides that, *Histoplasma* antigen detection has become consolidated the leading technique to DH diagnosis with excellent performance, easy to obtain specimens and also important for monitoring patients’ responses to antifungal therapy [28].

Our study has shown that more than a dozen publications reported the good performance of molecular methods in patients with DH, either alone or in association with conventional diagnostic methods (i.e., microscopy, culture and histopathology). Most of these studies have used whole blood, serum or fungal culture as the appropriate specimen for PCR testing, with a very high sensitivity and specificity. Molecular diagnosis can be effectively applied to lung biopsies, in situations in which other classical methods of pathogen identification are usually not effective [15,29]. Molecular tests are arguably important in regions where DH is not endemic, due to limited expertise in *H. capsulatum* identification.

The analytical performance of the molecular assays in the DH diagnosis varies according to disease stage and clinical form of histoplasmosis [30]. A recent meta-analysis showed the lack of consensus regarding the methodologies, particularly PCR protocols and gene targets (100-KDa-like-protein genes and amplification of ITS regions). For molecular assays, the overall specificity in the meta-analysis was 99% and overall sensitivity was 95%. Similar to the antigen testing, PCR presented excellent analytical performance, displaying a similar accuracy in comparison to the antigenuria-based assays [30]. By reviewing studies that compared primers, it was possible to see that Hcp100 is the most widely targeted gene and has shown better methodological performance [23,24].

This review demonstrated the performance of molecular tests in several studies involving patients with DH. PCR was applied to a wide range of clinical samples (e.g., biopsy, whole blood, serum, and cultures) [31,32]. The variability of molecular method applications is noticeable, which makes it difficult to make any relevant comparison. The reactions were done in different ways, with large variability in terms of DNA extraction, amplification targets, probes, and master mixes. No commercial PCR tests is available. In a multicenter comparison of PCR protocols for identification *H. capsulatum* DNA [24], all the protocols were specific and no false positive results were detected. Even though most studies in the literature have been based on nested PCR, real-time protocols were the most accurate and reliable. The applicability of PCR tests against Histoplasma species in the follow up of patients on treatment with antifungal drugs is also that deserve further investigation.

This study is limited by the scarce number of publications that directly compared immunological and molecular methods in patients with DH. There is a gap in the literature to be filled by future studies that consider this type of analysis, therefore contributing to the diagnosis of histoplasmosis. Most studies applied PCR from fungal cultures, which is of limited clinical interest.

In conclusion, this literature review showed that molecular tests have a good clinical performance in patients with DH. Most studies were performed with nested-PCR, so there is a need to further explore the application of real-time PCR in histoplasmosis. *Histoplasma* antigen detection by EIA has a central role in the DH diagnosis, but in only two publications these tests were compared to *Histoplasma* PCR. Considering that *Histoplasma* antigen is not commercially available in most endemic countries for histoplasmosis, and based on the assumption that many tertiary hospitals have PCR machines in place, molecular tests have the potential to facilitate the diagnostic routine of histoplasmosis in many circumstances.

## Figures and Tables

**Table 1:** Results of the literature review of studies in which molecular tests were used to diagnose disseminated histoplasmosis.

Author and Year	*n*	Samples	Molecular Technique	DNA Extraction	Molecular Target	Probe	Sensitivity	Specificity
Bialek R et al. (2002) [16]	100	Biopsy	Nested PCR	QIAamp tissue kit Qiagen	18S rDNA Hcp 100	None	NA	NA
Bracca, A. et al. (2003) [17]	30	Blood, biopsy, scraping from mucocutaneous lesions	Semi-nested PCR	Manual extraction	β-glucosidase	None	NA	NA
Guedes, H.L. et al. (2003) [18]	31	Fungal cultures	DNA Sequencing	Puregene DNA Kit Gentra.Systems	M Protein	None	100%	100%
Muñoz, C. et al. (2009) [19]	146	Fungal cultures	Nested PCR	DNA Minikit Qiagen	Hcp 100	None	100%	92%
Muñoz, B. et al. (2010) [20]	6	Fungal cultures	Conventional PCR	Manual extraction	M Protein	None	NA	NA
Frías-De-León, M. G. et al. (2012) [21]	40	Fungal cultures	(RAPD)-PCR	NA	SCAR Markers	None	NA	100%
Ohno, H. et al. (2013) [22]	7	Fungal cultures and cinical samples *	Nested PCR	NA	M Protein	None	NA	NA
Dantas, K. C. et al. (2013) [23]	40	Whole blood and serum	Nested PCR	NA	18S rDNA Hcp 100	None	96%–98%	HC100 (97%-Whole Blood, 95%-Serum) e HC18S (90% Whole Blood, 88% Serum)
Buitrago, M. J. et al. (2013) [24]	10	Fungal cultures	Nested, Real Time and Conventional PCR	DNeasy Plant MiniKit Qiagen	Hcp 100, SCAR, ITS1	Taqman HcITS-155	SCAR–43% Hcp 100 - 89.5	100%
Scheel, C. M. et al. (2014) [12]	50	Fungal cultures	Nucleic acid amplification via LAMP	Qiagen Dneasy Kit	Hcp100	None	NA	100%
Gago, S. et al. (2014) [25]	43	Fungal cultures and bronchoalveolar lavage	Multiplex real-time PCR	DNeasy Plant MiniKit Qiagen	ITS	None	90%	100%
Muraosa, Y. et al. (2015) [13]	19	Fungal cultures and clinical samples *	Cycling probe-based real-time PCR and Nested real-time PCR	PrepManTM Ultra Life Technologies	NAALADase Gene	Hist1probe3	Real Time PCR–77% Nested PCR–33%	NA
Brilhante, R. S. et al. (2016) [26]	16	Blood	Conventional PCR	CTAB	RYP1	None	100%	100%
Frías-De-León, M. G. et al. (2017) [11]	4	Serum	Nested PCR	Qiagen Dneasy Kit	Hcp100	None	NA	NA
Dantas, K.C. et al. (2018) [27]	40	Blood	Nested PCR	QIAamp Blood DNA Mini Kit	18S rDNA Hcp 100	None	18%–60%	85%–100%

Legend: NA, data not available; RAPD, random amplified polymorphic DNA; LAMP, Loop mediated isothermal amplification; PCR, Polymerase chain reaction; CTAB, cetyltrimethylammonium bromide. * Clinical Samples include blood, serum, biopsy, bronchoalveolar lavage, and scraping.

## References

[1] Chu J.H., Feudtner C., Heydon K., Walsh T.J., Zaoutis T.E. (2006). Hospitalizations for endemic mycoses: A population-based national study. Clin. Infect. Dis..

[2] Wheat L.J., Unlucky M.M., Bahr N.C., Spec A., Relich R.F., Hage C. (2016). Histoplasmosis. Infect. Dis. Clin. N. Am..

[3] Almeida M.A., Almeida-Silva F., Guimarães A.J., Almeida-Paes R., Zancopé-Oliveira R.M. (2019). The occurrence of histoplasmosis in Brazil: A systematic review. Int. J. Infect. Dis..

[4] Wheat L.J. (2006). Improvements in diagnosis of histoplasmosis. Expert Opin. Biol. Ther..

[5] Hage C.A., Ribes J.A., Wengenack N.L., Baddour L.M., Assi M., McKinsey D.S., Hammoud K., Alapat D., Babady N.E., Parker M. (2011). A multicenter evaluation of tests for diagnosis of histoplasmosis. Clin. Infect. Dis..

[6] Theel E.S., Jespersen D.J., Harring J., Mandrekar J., Binnicker M.J. (2013). Evaluation of an enzyme immunoassay for detection of *Histoplasma capsulatum* antigen from urine specimens. J. Clin. Microbiol..

[7] Costa M.R.E., Lacaz C.S., Kawasaki M., De Camargo Z.P. (2000). Conventional versus molecular diagnostic tests. Med. Mycol..

[8] Wheat J., Wheat H., Connolly P., Kleiman M., Supparatpinyo K., Nelson K., Bradsher R., Restrepo A. (1997). Cross-reactivity in *Histoplasma capsulatum* variety *capsulatum* antigen assays of urine samples from patients with endemic mycoses. Clin. Infect. Dis..

[9] Wheat L.J., Connolly P., Durkin M., Book B.K., Tector A.J., Fridell J., Pescovitz M.D. (2004). False-positive *Histoplasma* antigenemia caused by antithymocyte globulin antibodies. Transpl. Infect. Dis..

[10] LeMonte A., Egan L., Connolly P., Durkin M., Wheat L.J. (2007). Evaluation of the IMMY ALPHA *Histoplasma* antigen enzyme immunoassay for diagnosis of histoplasmosis marked by antigenuria. Clin. Vaccine Immunol..

[11] Frías-De-León M.G., Ramírez-Bárcenas J.A., Rodríguez-Arellanes G., Velasco-Castrejón O., Taylor M.L., Reyes-Montes M.R. (2017). Usefulness of molecular markers in the diagnosis of occupational and recreational histoplasmosis outbreaks. Folia Microbiol..

[12] Scheel C.M., Zhou Y., Theodoro R.C., Abrams B., Balajee S.A., Litvintseva A.P. (2014). Development of a Loop-Mediated Isothermal Amplification Method for Detection of *Histoplasma capsulatum* DNA in Clinical Samples. J. Clin. Microbiol..

[13] Muraosa Y., Toyotome T., Yahiro M., Watanabe A., Shikanai-Yasuda M.A., Kamei K. (2016). Detection of *Histoplasma capsulatum* from clinical specimens by cycling probe-based real-time PCR and nested real-time PCR. Med. Mycol. J..

[14] Tang Y.W., Li H., Durkin M.M., Sefers S.E., Meng S., Connolly P.A., Stratton C.W., Wheat L.J. (2006). Urine polymerase chain reaction is not as sensitive as urine antigen for the diagnosis of disseminated histoplasmosis. Diagn. Microbiol. Infect. Dis..

[15] Pineau S., Talarmin J.P., Morio F., Grossi O., Boutoille D., Léauté F., Le Pape P., Gay-Andrieu F., Miegeville M., Raffia F. (2010). Contribution de la biologie moléculaire et de l’antigénémie galactomannane aspergillaire au diagnostic de l’histoplasmose. Méd. Maladies Infect..

[16] Bialek R., Feucht A., Aepinus C., Just-Nübling G., Robertson V.J., Knobloch J., Hohle R. (2002). Evaluation of two nested PCR assays for detection of *Histoplasma capsulatum* DNA in human tissue. J. Clin. Microbiol..

[17] Bracca A., Tosello M.E., Girardini J.E., Amigot S.L., Gomez C., Serra E. (2003). Molecular detection of *Histoplasma capsulatum* var. *capsulatum* in human clinical samples. J. Clin. Microbiol..

[18] Guedes H.L., Guimarães A.J., Muniz M.d.M., Pizzini C.V., Hamilton A.J., Peralta J.M., Deepe G.S.J., Zancopé-Oliveira R.M. (2003). PCR assay for identification of *Histoplasma capsulatum* based on the nucleotide sequence of the M antigen. J. Clin. Microbiol..

[19] Muñoz C., Gómez B.L., Tobón A., Arango K., Restrepo A., Correa M.M., Muskus C., Cano L.E., González A. (2009). Validation and clinical application of a molecular method for identification of *Histoplasma capsulatum* in human specimens in Colombia, South America. Clin. Vaccine Immunol..

[20] Muñoz B., Martínez M.A., Palma G., Ramírez A., Frías M.G., Reyes M.R., Taylor M.L., Higuera A.L., Corcho A., Manjarrez M.E. (2010). Molecular characterization of *Histoplasma capsulatum* isolated from an outbreak in treasure hunters *Histoplasma capsulatum* in treasure hunters. BMC Infect. Dis..

[21] Frías De León M.G., Arenas López G., Taylor M.L., Acosta Altamirano G., Reyes-Montes M.R. (2012). Development of specific sequence-characterized amplified region markers for detecting *Histoplasma capsulatum* in clinical and environmental samples. J. Clin. Microbiol..

[22] Ohno H., Tanabe K., Umeyama T., Kaneko Y., Yamagoe S., Miyazaki Y. (2013). Application of nested PCR for diagnosis of histoplasmosis. J. Infect. Chemother..

[23] Dantas K.C., Freitas R.S., Moreira A.P., Silva M.V., Benard G., Vasconcellos C., Criado P.R. (2013). The use of nested Polymerase Chain Reaction (nested PCR) for the early diagnosis of *Histoplasma capsulatum* infection in serum and whole blood of HIV-positive patients. An. Bras. Dermatol..

[24] Buitrago M.J., Canteros C.E., Frías De León G., González Á., Marques-Evangelista De Oliveira M., Muñoz C.O., Ramirez J.A., Toranzo A.I., Zancope-Oliveira R., Cuenca-Estrella M. (2013). Comparison of PCR protocols for detecting *Histoplasma capsulatum* DNA through a multicenter study. Rev. Iberoam. Micol..

[25] Gago S., Esteban C., Valero C., Zaragoza O., Puig de la Bellacasa J., Buitrago M.J. (2014). A multiplex real-time PCR assay for identification of *Pneumocystis jirovecii*, *Histoplasma capsulatum*, and *Cryptococcus neoformans*/*Cryptococcus gattii* in samples from AIDS patients with opportunistic pneumonia. J. Clin. Microbiol..

[26] Brilhante R.S., Guedes G.M., Riello G.B., Ribeiro J.F., Alencar L.P., Bandeira S.P., Castelo-Branco D.S., Oliveira J.S., Freire J.M., Mesquita J.R. (2016). RYP1 gene as a target for molecular diagnosis of histoplasmosis. J. Microbiol. Methods.

[27] Dantas K.C., Freitas R.S., Silva M.V., Criado P.R., Luiz O.D.C., Vicentini A.P. (2018). Comparison of diagnostic methods to detect *Histoplasma capsulatum* in serum and blood samples from AIDS patients. PLoS ONE.

[28] Theel E.S., Harring J.A., Dababneh A.S., Rollins L.O., Bestrom J.E., Jespersen D.J. (2015). Reevaluation of commercial reagents for detection of *Histoplasma capsulatum* antigen in urine. J. Clin. Microbiol..

[29] Falci D.R., Hoffmann E.R., Paskulin D.D., Pasqualotto A.C. (2017). Progressive disseminated histoplasmosis: A systematic review on the performance of non-culture-based diagnostic tests. Braz. J. Infect. Dis..

[30] Caceres D.H., Knuth M., Derado G., Lindsley M.D. (2019). Diagnosis of Progressive Disseminated Histoplasmosis in Advanced HIV: A Meta-Analysis of Assay Analytical Performance. J. Fungi.

[31] Imhof A., Schaer C., Schoedon G., Schaer D.J., Walter R.B., Schaffner A., Schneemann M. (2003). Rapid detection of pathogenic fungi from clinical specimens using LightCycler real-time fluorescence PCR. Eur. J. Clin. Microbiol. Infect. Dis..

[32] Martagon-Villamil J., Shrestha N., Sholtis M., Isada C.M., Hall G.S., Bryne T., Lodge B.A., Reller L.B., Procop G.W. (2003). Identification of *Histoplasma capsulatum* from culture extracts by real-time PCR. J. Clin. Microbiol..

